# The Impact of the COVID-19 Pandemic on Consumer Behavior—Evidence From China's Stock Market

**DOI:** 10.3389/fpubh.2022.865470

**Published:** 2022-09-06

**Authors:** Zhou Lu, Linchuang Zhu, Xiaoxin Li, Zhenhui Li

**Affiliations:** ^1^Qingdao City University, Qingdao, China; ^2^Tianjin University of Commerce, Tianjin, China; ^3^Guangdong University of Foreign Studies, Guangzhou, China; ^4^Communication University of China, Beijing, China

**Keywords:** COVID-19, stock market, China, retail industry, consumer behavior

## Abstract

The COVID-19 pandemic has dramatically reshaped consumers' grocery shopping behavior. Meanwhile, change in consumer shopping behavior might further exert a considerable and far-reaching impact on the food retail industry. Although the existing literature provides investigation on the impact of the pandemic on the retail industry, very few studies discuss the impact of changes in consumer shopping behavior on the stock market performance of the retail industry. This paper investigates selected food retailers listed in China's stock market. To overcome the problems of the Chow test, the Quandt-Andrews test was used to identify the dates of breakpoints of structural change in the stock price performance of those selected companies. The results suggest that there has indeed been an industry-wide structural change in the stock market performance during the pandemic. The study found that the dates of breakpoints for the selected companies were concentrated in the first half of 2020, when China was hit by the Covid-19 pandemic the most. Our survey shows that under strict epidemic prevention and control measures, consumers have gradually adapted to the new normal of epidemic prevention to a certain extent, established safety awareness, and changed their consumption behavior. Our study on stock price data implies that Chinese consumers experienced a shift from physical store offline purchases to online purchasing model.

## Introduction

The COVID-19 pandemic has exerted a tremendous impact on the global economy^1^,^2^ (1). During the pandemic, China took strict measures such as travel restriction and community “lock-down” to fight the epidemic. Those measures may have reshaped the behavior of billions of Chinese people. A remarkable change took place in the shopping behavior of consumers in China as they have greatly reduced exposure to the public, especially places such as supermarkets and shopping malls^3^,^4^. With the restaurant shut-down wave in China during the epidemic, many people in China, especially young consumers, have to rely on home cooking. Hence, the demand of Chinese households for fresh products (1) and packaged food have experienced substantial growth. In addition, consumers used to shop at physical retail stores rather than online platforms before the epidemic. By contrast, during the epidemic, they tend to shift from offline purchase to online purchase.

The change in Chinese consumers' shopping behavior driven by the epidemic is also considered to be projected on the stock market. Investigation of the impact of major public health events such as the “COVID-19” on consumers' shopping behavior helps the food industry improve quality and efficiency and promote innovations in operation and marketing. This paper selects the food-based retail industry to study whether the “COVID-19” pandemic leads to a change in consumer behavior using data from the stock market.

## Literature Review

Compared with other grocery goods, fresh produce goods are perishable and require good quality cold chain transportation^5^–^10^ (2, 3). With the outbreak of the Covid-19 pandemic, traffic control has been introduced across China. Many scholars have carried out research on this control from the perspective of supply chain in agricultural products. Wenjin (4) analyzed the serious impact of the outbreak on the supply chain structure of agricultural products from the perspective of supply risk, demand risk, safety risk and environmental risk. Xicai (5) analyzed the impact of the epidemic on the supply chain of agricultural products from the perspectives of supply and demand. He pointed out that the sudden outbreak of the epidemic has greatly changed the consumption habits and lifestyles of residents and online grocery shopping and home delivery is the trend. Yanan (6) investigates the impact of social responsibility performance on the performance improvement in the e-commerce supply chain of fresh produce under public health emergencies. Gucheng (7) analyzed the impact of the epidemic on the agricultural product supply chain in Wuhan before and after the “city lock-down.” The study found that the “city lock-down” measure has exacerbated the impact of the epidemic on the agricultural product supply chain, and the impact of this measure on different types of agricultural product suppliers varies. Mitchell et al. (8) examine the response of the U.K. fruit and vegetable food supply chain to COVID-19, and their findings show that, despite major disruptions, the retail-led fresh food supply chain has shown a high degree of resilience.

In addition to the research from the perspective of supply chain, some scholars have also explored the impact of the epidemic on the agricultural product industry from other perspectives. Chen et al. (9) studied the impact of the epidemic on consumers' fresh food purchasing behavior, and the results showed that more citizens buy fresh food online. Butu et al. (10) also explored the direct impact of the COVID-19 crisis on consumers' fresh vegetable purchasing behavior.

Although there have been some discussions on the impact of the “COVID-19” pandemic in the existing literature, there is little literature on the impact of the epidemic on consumers shopping behavior from the perspective of the stock market. Therefore, this paper selects representative retail companies listed on China's stock market and investigates the impact of the epidemic on the stock prices of those companies. The economic impact of the shock of the COVID-19 pandemic on the grocery retail industry is examined. The Quandt-Andrews test is used to identify the structural change in the stock price during the epidemic for each selected company. Furthermore, based on the structural changes of those companies, we analyze whether the epidemic has changed consumer shopping behavior to expand the existing research.

## Methodology, Data and Empirical Results

The outbreak of the Covid-19 pandemic has brought an unprecedented impact on the food retail industry, which is manifested by the structural changes in the stock prices of representative retailers from the industry. In order to identify the breakpoints of structural change in the stock price data, this paper conducts parameter stability test on the stock price time series data for each selected representative food retail company in China. The parameter stability test, also known as the structural change test, aims to test for the change in the structural parameters of a model. However, the breakpoint date of a structural change in parameters is often unknown. In view of the shortcomings of Chow test, Quandt and Andrews proposed a test method for unknown breakpoints of structural change. One or more break points may exist on the value interval (τ1, τ2). According to the Quandt-Andrews method, the interval (τ 1, τ 2) is first divided into *k* subintervals, and the Chow test is made for each subinterval. Then, those *k* statistics from Chow test are summarized into one single statistic to test for a structural change between τ1 and τ2.There are three ways to summarize the *k* statistics from Chow test into the statistic of the Quandt-Andrews test: (1) *Maximum* statistic, the maximum value of the *k* statistics; (2) *Ave* statistic, the simple arithmetic mean of the *k* statistics; (3) *Exp* statistic defined in equation (3).

*Max* statistic is defined as the following equation:


(1) $$\begin{array}{r} \max F=\max_{\tau_{1}\leq \tau \leq \tau_{2}}F\left(\tau \right) \end{array}$$

*Ave* statistic is the simple average of the *k* individual statistics:


(2) $$\begin{array}{r} AveF=\frac{1}{k}\overset{\tau_{2}}{\underset{\tau =\tau_{1}}{\sum }}F\left(\tau \right) \end{array}$$

*Exp* statistics is shown in the following equation:


(3) $$\begin{array}{r} \exp F=\ln\;\left(\frac{1}{k}\overset{\tau_{2}}{\underset{\tau =\tau_{1}}{\sum }}\exp\left(\frac{1}{2}F\left(\tau \right)\right)\right) \end{array}$$

In order to effectively identify the breakpoints of structural change in the stock price data for the selected representative food retailer in China's stock market, this paper selects the time series of the average daily stock prices from 2019 to 2022 for empirical analysis.

In terms of the selection of food retailers, the listed companies with the main revenue source from the food retail industry as shown by their annual reports are selected. The food retail revenue accounts for more than 50% of the total revenue. In order to identify the changes in the company's stock price before and after the epidemic, our data are based the daily average price data for five selected food retailer for a total of 803 days from November 1, 2019 to January 5, 2022. The selected companies are Jiajiayue Group Co., Ltd. (hereinafter referred to as Jiajiayue), Yonghui Superstores Co., Ltd. (hereinafter referred to as Yonghui Supermarket), Sanjiang Shopping Club Co.,Ltd (hereinafter referred to as Sanjiang Shopping), Renrenle Commercial Group Co., Ltd. (hereinafter referred to as Renrenle), and Rainbow Digital Commercial Co., Ltd. Co., Ltd. (Rainbow Digital Commercial Co., ltd.) (hereinafter referred to as Rainbow). Stock price data are all from the Wind database.

Based on the above time series data, the Quandt-Andrews test is used to test the parameter stability for the breakpoints of structural change, and the statistic size of Equations (1)–(3) is calculated to obtain the approximate asymptotic *p*. The test results are shown in Table 1. Note that for each individual Chow test, two types of F statistics can be obtained, that are *Likelihood Ratio F*-statistic and *Wald* F-statistic. Hence, the result of the above three Quandt-Andrews statistics can be presented either in *LR F-*statistic value or *Wald F-*statistic value.

**Table 1:** The results of Quandt-Andrews test for selected food retailer listed in China's stock market.

**company name**	**Mutation date**	**Statistics**		***P*-value**
Jiajiayue	5/27/2020	Maximum LR F-statistic	233504.2	0.000
		Maximum Wald F-statistic	467008.5	0.000
		Exp LR F-statistic	116746.2	0.000
		Exp Wald F-statistic	233498.3	0.000
		Ave LR F-statistic	−43.49353	0.000
		Ave Wald F-statistic	−86.98707	0.000
Yonghui Supermarket	4/02/2020	Maximum LR F-statistic	59579.08	0.000
		Maximum Wald F-statistic	119158.2	0.000
		Exp LR F-statistic	29783.62	0.000
		Exp Wald F-statistic	59573.16	0.000
		Ave LR F-statistic	−666.6386	0.000
		Ave Wald F-statistic	−1333.277	0.000
Sanjiang Shopping	5/15/2020	Maximum LR F-statistic	581677.5	0.000
		Maximum Wald F-statistic	1163355.0	0.000
		Exp LR F-statistic	290832.8	0.000
		Exp Wald F-statistic	581671.6	0.000
		Ave LR F-statistic	−10131.05	0.000
		Ave Wald F-statistic	−20262.09	0.000
Everyone is happy	4/09/2020	Maximum LR F-statistic	4391152.0	0.000
		Maximum Wald F-statistic	8782303.0	0.000
		Exp LR F-statistic	2195570.0	0.000
		Exp Wald F-statistic	4391146.0	0.000
		Ave LR F-statistic	5965.736	0.000
		Ave Wald F-statistic	11931.47	0.000
Rainbow shares	4/14/2020	Maximum LR F-statistic	1806.334	0.000
		Maximum Wald F-statistic	3612.668	0.000
		Exp LR F-statistic	897.2481	0.000
		Exp Wald F-statistic	1800.415	0.000
		Ave LR F-statistic	−300.2362	0.000
		Ave Wald F-statistic	−600.4724	0.000

Table 1 shows the Quandt-Andrews test results of selected food retailers. It can be seen that at the 1% significance level, the Quandt-Andrews maximum statistics, *Exp* statistics and *Ave* statistics of each company all have passed the significance test, indicating that there exist breakpoints of structural change in all companies.

The maximum statistic > *Exp* statistic > *Ave* statistic.

In terms of the dates of the breakpoints, it can be found that the three companies, Yonghui Supermarket, Renrenle, and Rainbow Co., Ltd., have undergone structural change in April 2020. The breakpoint dates occurred on April 02, 2020, April 09, 2020, and April 14, 2020, respectively in less than half month. And the other two companies, Sanjiang Shopping and Jiajiayue, underwent structural change in May 2020. The breakpoint dates were May 15, 2020 and May 27, 2020. These two breakpoint dates were nearly 1 month behind Yonghui Supermarket, Renrenle, and Rainbow. However, the breakpoint dates of these five companies all occurred in the first half of 2020.

Feiteng (11) believes that China has entered the “post-epidemic era” in June 2020. As of 24:00 June 30, 2020, a total of 83,534 confirmed cases have been reported in mainland China, and 1,698 confirmed cases have been reported in Hong Kong, Macao and Taiwan [National Health Commission of the People's Republic of China (11)]. During the second half of 2020, there were 3,537 new confirmed cases in mainland China and 7,993 new confirmed cases in Hong Kong, Macao and Taiwan (National Health Commission of the People's Republic of China) (2). From 2021 to January 2, 2022, there were 15,595 new confirmed cases in mainland China, and 20,122 new confirmed cases in Hong Kong, Macao and Taiwan (National Health Commission of the People's Republic of China) (3). Hence, the first half of 2020 was obviously the period hit by the epidemic the most in China. Meanwhile, all selected food retailers are shown to experienced structural changes during the first half of 2020.This result might imply the change in Chinese consumers' shopping behavior because Chinese consumers used to rely on physical retail stores and they experienced substantial shift to online purchases driven by the epidemic.

During the outbreak of the epidemic in early 2020, the Chinese government responded to this public health emergency quickly. Consumers across China actively responded to the call for stringency and restrictions on public gathering. During the Chinese New Year 2020, the shut-down of a large number of restaurants in China also affected many young people. They started cooking at home. Driven by the epidemic prevention policies, online purchase of fresh food has become the first choice for many of them. The emergence of these factors has led consumers to prefer to buy fresh food online. At the beginning of 2020, the sales volume of fresh food e-commerce platforms such as Daily Youxian, JD Daojia, Dingdong Buying Foods and Fresh Hema all experienced a remarkable surge of 220−470% during the same period. Consumers' attempts to purchase fresh food online in the early stage of the epidemic have cultivated the habits of on-line shopping. In the post-epidemic era, the model of fresh food e-commerce has been widely accepted by Chinese consumers. With the rapid development of the Internet and the improvement of the logistics system, fresh food e-commerce will become an important business model to satisfy consumers' grocery needs. Therefore, such consumers who buy fresh food online have changed their consumption behavior after the epidemic. Instead of shopping at a physical off-line store offline, they tend to turn to online purchases.

## Conclusions

This paper examines the impact of the “COVID-19” pandemic on the food retail industry from the perspective of stock prices in capital market. The high-frequency daily stock price data of selected food retailers were analyzed to investigate if the COVID-19 pandemic has exerted impact on the food retail industry. The results show that the stock prices experienced significant structural change for all 5 selected food retailers in China. In addition, for all selected retailers, the dates of structural change are all shown to take place in the first half of 2020 when China was hit by the Covid-19 pandemic with the highest intensity.

The impact of the Covid-19 pandemic on the stock market performance of food retail industry might imply a substantial change in Chinese consumers' shopping behavior. Our study provides evidence which implies a significant shift of Chinese consumers' grocery shopping behavior from physical stores to online purchases. Consumers' favorable attitude to online fresh food purchase make fresh food e-commerce platform an important model to satisfy consumers' grocery needs. Therefore, consumers who buy fresh food online have changed their consumption behavior after the epidemic. Instead of relying on offline purchases, they tend to move to online.

However, there is still room for a considerable growth in fresh food e-commerce platform in China. The “Covid-19” pandemic, to a certain extent, promotes the customer base of fresh food e-commerce platform. Our study also suggests that food retail companies should make reasonable improvements, reduce logistics costs & operating costs, and increase total revenue. Given the emergence of the Big Data era, the food retailers can use offline physical stores as the operating portal, and use physical store operations to improve product visibility and customer traffic. Food retail stores combined with online sale can promote the integration of online business and offline business in food industry, thereby promoting the overall performance of the whole industry. In addition, the food retail industry can better adapt to the change in consumer behavior by improving the supply capacity of food products in the context of big data.

## Data Availability Statement

The original contributions presented in the study are included in the article/supplementary material, further inquiries can be directed to the corresponding author/s.

## Author Contributions

ZLu and ZLi: conceptualization. ZLu: formal analysis and funding acquisition. LZ and XL: methodology and data processing. LZ and ZLu: writing—original draft and revision. ZLi: project management. All authors contributed to the article and approved the submitted version.

## Funding

The authors acknowledge the financial supports from the Philosophy and Social Science Fund of Tianjin City, China (Award #: TJYJ20-012).

## Conflict of Interest

The authors declare that the research was conducted in the absence of any commercial or financial relationships that could be construed as a potential conflict of interest.

## Publisher's Note

All claims expressed in this article are solely those of the authors and do not necessarily represent those of their affiliated organizations, or those of the publisher, the editors and the reviewers. Any product that may be evaluated in this article, or claim that may be made by its manufacturer, is not guaranteed or endorsed by the publisher.

## Footnotes

^1^Food refers to fresh products and packaged foods, the same below.

^2^Fresh products generally refer to unprocessed primary agricultural products, including vegetables, fruits, meat, eggs, milk, and aquatic products.

^3^Food refers to fresh and cooked food and packaged food.

^4^Zhong Feiteng. Slow globalization: New crown epidemic and China's external economic environment [J]. Teaching and Research, 2020(10): 69-80.

^5^ http://www.nhc.gov.cn/yjb/s7860/202007/a98e49570be24eaf88de98e6e6217fc8.shtml

^10^China's New Economy Investment Research Report 2020 [C]. Iresearch Series Research Report (the third issue of 2020), 2020:158-208.
